# Association between timing and number of antenatal care visits on uptake of intermittent preventive treatment for malaria during pregnancy among Malawian women

**DOI:** 10.1186/s12936-018-2360-z

**Published:** 2018-05-25

**Authors:** Owen Nkoka, Ting-Wu Chuang, Yi-Hua Chen

**Affiliations:** 1 School of Public Health, College of Public Health, Taipei Medical University, Taipei, Taiwan; 2 Department of Molecular Parasitology and Tropical Diseases, School of Medicine, College of Medicine, Taipei Medical University, Taipei, Taiwan

**Keywords:** Antenatal attendance, Intermittent preventive treatment, Malaria in pregnancy, Malawi

## Abstract

**Background:**

Malaria in pregnancy is a critical public health challenge, and intermittent preventive treatment for malaria during pregnancy (IPTp) has proven to be an effective intervention. However, access to and use of malaria interventions, including IPTp, remains a considerable problem among African women. This cross-sectional study investigated factors, including antenatal care (ANC) attendance (both numbers of visits and timing of the first visit) and socio-demographics, associated with the uptake of the recommended IPTp dose among Malawian women.

**Methods:**

A nationally representative sample of women with a live birth in the 2 years preceding the survey from the Malawi Demographic Health Survey 2015–2016 dataset was analysed. Self-reported data on socio-demographics, ANC attendance and IPTp uptake were collected using a questionnaire and analysed using logistic models.

**Results:**

Of the 6549 included women, 1981 (30.2%) took the recommended three or more IPTp doses. Despite inadequate ANC visits, early ANC initiation increased the likelihood of these women taking the recommended IPTp dose; women who initiated ANC in the first [adjusted odds ratio (aOR) = 2.24; 95% confidence interval (CI) = 1.44–3.49] and second (aOR = 2.19; 95% CI = 1.56–3.08) trimesters were more likely to take the recommended IPTp dose compared to late initiators. The effect of the number of ANC visits on IPTp uptake was significant in married women (aOR = 1.68, 95% CI = 1.42–1.98), and the timing of first ANC visit was associated with IPTp uptake only among rural women (aOR = 2.13, 95% CI = 1.54–2.95).

**Conclusion:**

ANC attendance is vital in IPTp uptake. The results highlight the need for health care providers to encourage women, particularly those in high-risk groups, to make frequent ANC visits and receive early ANC initiation to ensure high coverage of the recommended IPTp dose.

## Background

Malaria remains a critical public health issue, with particular burden of disease on children aged younger than 5 years and pregnant women [1]. Malaria in pregnancy (MIP) poses a serious threat to mothers, fetuses and newborns, leading to infant and maternal mortality, low birth weight, and maternal anaemia [1]. Globally, MIP is responsible for 10,000 maternal deaths per year [2]. Furthermore, in sub-Saharan Africa, it accounts for 20 and 11% of stillborn and neonatal deaths, respectively [3, 4].

Malawi is a malaria-endemic country. Between 2009 and 2014, an infection rate of 19.6% was reported among pregnant women attending their first antenatal care (ANC) service in a peri-urban area of Ndirande, in Blantyre [5]. In addition to the aforementioned impact of MIP, the economic burden of malaria in Malawi is high. An average Malawian family spends approximately US$17.48 per malaria episode, which is higher than a week’s income for most Malawian families [6]. Considering the adverse effects of MIP, the use of intermittent preventive treatment for malaria during pregnancy (IPTp) is one of the most important strategies adopted by the Malawi Ministry of Health (MoH) to reduce the MIP burden [7].

In IPTp, pregnant women are prescribed sulfadoxine–pyrimethamine (SP) [8]. The World Health Organization (WHO) recommends that women in malaria-endemic areas should be given the IPTp dose during ANC visits as early as possible in the second trimester and that subsequent doses should be given at 4-week intervals [9]. An appropriate dose of IPTp reduces maternal mortality along with the risks of maternal anaemia and low birth weight [10]. A Malawian study, conducted in Blantyre from July 1997 to April 1999, reported a significant association between SP use and reduced placental malaria parasite prevalence among IPTp non-users (31.9%) and users (22.8%) [11].

In 1993, Malawi became the first country worldwide to implement an IPTp policy, recommending two doses of SP to all pregnant women [12–14], 5 years before the WHO released its IPTp recommendations [15]. Thereafter, the coverage with two doses of IPTp (formerly, the recommended dose) has been relatively high in Malawi compared to the rest of sub-Saharan Africa [16–18]. In 2015, 63% of pregnant women received at least two doses; the percentage of women receiving one IPTp dose increased from 80.7% in 2006 to 89% in 2015 [19, 20]. In Malawi, monitoring of the uptake of 3 IPTp doses started in 2014, after IPTp policy was updated by the WHO in 2012 [9, 19]; however, the uptake of 3 doses was as low as 12% in 2014. Although the 3-dose recommendation was new in 2014 and despite it being a regional leader in IPTp uptake, Malawi still failed to meet its own 2011–2015 Malaria Strategic Plan target of having 80% coverage of the uptake of 2 IPTp doses among pregnant women [18, 19, 21]. Nevertheless, increasing its uptake is crucial to reducing MIP burden. The effects of socio-demographics (e.g., education and parity) on IPTp uptake have been well documented [22–27]. For instance, a 2009 study conducted in 8 Malawian districts revealed that women who had secondary- or tertiary-level education were more likely to take the recommended number of IPTp doses than those without formal education [24]. However, studies have also reported contrasting findings regarding the influence of socio-demographics on IPTp uptake [22–25, 27]. Although a Tanzanian study found parity to be a crucial predictor of IPTp uptake [23], no association was reported in western Nigeria [28].

Although several studies have examined the effects of ANC attendance on IPTp uptake, the association has been inconsistent [26, 29–33]. For example, a Kenyan study found an association between the timing of first ANC visit and uptake of IPTp [29], whereas studies in Uganda, Tanzania, and Nigeria have reported no such association [34–37]. In Cameroon, less frequent clinic visits during pregnancy prevented the uptake of the recommended IPTp dose [38], whereas no such association was observed in Kenya [29]. The observed differences might be due to the various assessment durations; the Kenyan study [29] included not only currently pregnant women but also mothers with children aged under 1 year, whereas the Cameroonian study [38] was restricted to pregnant women. In addition, this inconsistency might be explained by the lack of consideration of the potential interactive effects of socio-demographics and ANC attendance on IPTp uptake.

Understanding the factors affecting IPTp uptake is critical for promoting and designing targeted interventions to improve the coverage of IPTp. Studies on the influence of both ANC attendance and socio-demographics on IPTp have focused only on their independent effects; thus far, limited empirical evidence is available on their interactive effects. Examining the effect modifiers of the relationship between ANC attendance and IPTp uptake is critical for identifying groups with a high risk of low IPTp uptake. Furthermore, although both the number of ANC visits and timing of first ANC visit are prominent factors, studies on ANC attendance and IPTp uptake have not elucidated how these two factors (i.e., number and timing) independently affect IPTp uptake, considering that the timing of first ANC visit may also influence the number of ANC visits, as reported in Tanzania [39].

Therefore, this study examined the relationship between ANC attendance, socio-demographics, and IPTp uptake among Malawian women by using a nationally representative sample. In particular, the study aimed to: (1) examine the factors (i.e., ANC attendance and socio-demographics) associated with the uptake of the recommended IPTp dose; (2) evaluate the independent association between the number of ANC visits and timing of first ANC visit on IPTp uptake; and, (3) investigate the moderating effects of socio-demographics on the relationship between ANC attendance and IPTp uptake.

## Methods

### Study design and data source

This cross-sectional study utilized data from the 2015 to 2016 Malawi Demographic and Health Survey (MDHS) [40]. The study design, methodology and sampling method have been described elsewhere in detail [40]. In summary, the sampling frame was the 2008 Malawi Population and Housing Census [40]. A two-stage stratified sampling method was used. The first stage involved the selection of standard enumeration areas (SEAs) from among 850 SEAs of Malawi. The second stage involved equal probability systematic selection of households in each SEA. Women aged 15–49 years who were either permanent residents of the selected households or visitors who stayed in the household the night before the survey were eligible to be interviewed. Data were collected from 9 October, 2015 to 17 February, 2016. The questionnaire was translated into Chichewa and Tumbuka (prominent local languages). Pre-testing was conducted in July and August 2015. All data collectors were trained and had experience with household surveys. Of 25,146 eligible women, 24,562 were successfully interviewed, indicating a 98% response rate.

To reduce recall bias and in line with the Roll Back Malaria Indicator on IPTp, the sample was restricted to women who had had a live birth in the 2 years preceding the survey [27, 41]. In total, 6549 women were finally included.

### Outcome variable

As per WHO recommendations, the outcome measure was defined as the uptake of fewer than 3 IPTp doses (low uptake) or the uptake of 3 or more IPTp doses (recommended dose) [9].

### Independent variables

ANC attendance, the main independent predictor for IPTp uptake in this study, was assessed using two items, namely ‘number of ANC visits’ and ‘timing of first ANC visit’ [25, 38]. The number of ANC visits was categorized into two levels: adequate (4 or more) and inadequate (fewer than 4), according to WHO recommendations [9, 42]. The timing of first ANC visit was categorized according to the first, second, and third trimester.

Socio-demographics were also examined for their effects on IPTp uptake. In particular, three-level variables were used to categorize age (15–24, 25–34 and ≥ 35 years), education (no formal education, primary education, secondary and above), marital status (married, never married, separated, widowed or divorced), and parity (primigravida, secundigravida, multigravida). Marital status was further assessed as a binary variable (married *vs* unmarried) in sub-group analysis. Principal component analysis was applied to the MDHS data for calculating wealth on the basis of household assets, such as bicycles and mobile phones. The calculated wealth was then categorized into quintiles, with the lowest to highest indicating the poorest to richest. In this study, wealth was categorized into a three-level variable by combining two sub-categories into single categories: poorest and poor into poor and rich and richest into rich, with the middle category unchanged.

### Statistical analyses

The distribution of participants according to the number of ANC visits was analysed using the Chi square test. The distributions of ANC attendance and socio-demographics by IPTp uptake, along with their effects on the uptake, were further examined.

To investigate the association between the number of ANC visits and timing of first ANC visit on IPTp uptake, a single variable, ANC use, was generated and coded into the following 6 groups: (1) inadequate ANC visits and first ANC visit in first trimester; (2) inadequate ANC visits and first ANC visit in second trimester; (3) inadequate ANC visits and first ANC visit in third trimester; (4) adequate ANC visits and first ANC visit in first trimester; (5) adequate ANC visits and first ANC visit in second trimester; and, (6) adequate ANC visits and first ANC visit in third trimester. Group 3 (inferior situation) was used as the reference group, against which women from the other categories were compared. Logistic regression analyses were performed to estimate ORs and their 95% CIs for the association between ANC use and recommended IPTp uptake.

Interactions of ANC attendance (i.e., number of ANC visits and timing of first ANC visit) and socio-demographics with IPTp uptake were examined. A *p* of < 0.1 was used for interaction terms to signal potential moderation [43], and a sub-group analysis was further used to examine the moderation effects.

All analyses considered the complex sample design, and only weighted data were used for analysis. Sample weighting allowed for adjustments to the cluster sampling design and sampling probabilities across clusters and strata. The level of significance was set at *p *< 0.05 (two-tailed), and all analyses were performed using SPSS software version 22.0 (SPSS, Chicago, IL, USA).

### Ethical considerations

Informed consent for the survey was obtained from each respondent at the start of each individual interview. Ethical approval was obtained from the Malawi National Health Sciences Research Committee of the Malawi Ministry of Health prior to the survey. The MDHS data sets are publicly available, and clearance to analyse them was provided by the International Classification of Functioning Disability and Health (ICF) under the Demographic Health Survey (DHS) programme.

## Results

### Distribution of participants by number of ANC visits

In total, 6549 women with a live birth in the 2 years prior to the survey participated in this study. Of them, 48.1% had adequate ANC visits. Table 1 displays the distribution of participants according to their number of ANC visits. The difference between women with adequate ANC visits and with inadequate ANC visits was significant (*p *< 0.05), in terms of age, marital status, education, occupation, wealth, residence, IPTp uptake, and timing of first ANC visit, but not parity and region.

**Table 1:** Distribution of participants according to number of ANC visits

Variable	Total (*n *= 6549)	Number of ANC visit categories		*p* value^b^
		Inadequate^a^ (*n *= 3402)	Adequate^a^ (*n *= 3147)	
	*n*	*n* (%)	*n* (%)	
Age (years)				0.035*
15–24	3043	1642 (48.3)	1401 (44.5)	
25–34	2517	1246 (36.6)	1271 (40.4)	
≥ 35	989	514 (15.1)	475 (15.1)	
Marital status				0.012*
Married	5470	2799 (82.3)	2671 (84.9)	
Never married	346	210 (6.2)	136 (4.3)	
Separated/divorced/widowed	733	393 (11.6)	340 (10.8)	
Education				< 0.001***
No formal education	785	444 (13.0)	341 (10.8)	
Primary	4382	2350 (69.1)	2032 (64.6)	
Secondary and above	1382	608 (17.9)	774 (24.6)	
Occupation				0.049*
Unemployed	2086	1130 (33.2)	956 (30.4)	
Employed	4463	2272 (66.8)	2191 (69.6)	
Parity				0.132
Primigravida	1818	899 (26.4)	919 (29.2)	
Secundigravida	1352	722 (21.2)	630 (20.0)	
Multigravida	3379	1781 (52.4)	1598 (50.8)	
Wealth index				< 0.001***
Poor	3147	1724 (50.7)	1423 (45.2)	
Middle	1261	690 (20.3)	571 (18.2)	
Rich	2141	988 (29.0)	1153 (36.6)	
Residence				< 0.001***
Urban	897	381 (11.2)	516 (16.4)	
Rural	5652	3021 (88.8)	2631 (83.6)	
Region				0.841
Northern	748	389 (11.4)	359 (11.4)	
Central	2756	1418 (41.7)	1338 (42.5)	
Southern	3045	1595 (46.9)	1450 (46.1)	
IPTp uptake				< 0.001***
< 3	4568	2551 (75.0)	2017 (64.1)	
≥ 3	1981	850 (25.0)	1131 (35.9)	
Timing of first ANC visit (*n *= 6413)				< 0.001***
First trimester	1503	325 (9.9)	1178 (37.5)	
Second trimester	4427	2492 (76.2)	1935 (61.6)	
Third trimester	483	452 (13.8)	31 (1.0)	

*ANC* antenatal care, *IPTp* intermittent preventive treatment for malaria during pregnancy

* *p* < 0.05; ** *p* < 0.01; *** *p* < 0.001

^a^The number of ANC visits was categorized into two levels: adequate ANC visits (4 or more) and inadequate ANC visits (fewer than 4)

^b^*p* value of Pearson’s Chi squared test

### Distribution of participants by IPTp uptake and potential predictors recommended IPTp uptake

Approximately 30.2% of the included women received the recommended IPTp dose. Distributions of potential predictors of IPTp uptake are listed in Table 2. Significant differences were observed between those with and without recommended IPTp uptake (*p *< 0.05), in terms of region, number of ANC visits, and timing of first ANC visit, but not the remaining variables. In the crude logistic regression analysis, age of ≤ 24 years (OR = 1.25, 95% CI = 1.03–1.51), adequate ANC visits (OR = 1.68, 95% CI = 1.47–1.92), and first ANC visit in first (OR = 2.72, 95% CI = 1.97–3.76) and second (OR = 2.30, 95% CI = 1.69–3.12) trimesters were significantly associated with increased odds of taking the recommended IPTp dose (*p* < 0.05).

**Table 2:** Distribution of participants by uptake of IPTp and potential factors affecting uptake

Variable	< 3 doses (*n *= 4568)	≥ 3 doses (*n *= 1981)	*p* value^a^	OR	(95% CI)
	*n* (%)	*n* (%)			
Age (years)			0.055		
15–24	2070 (45.3)	973 (49.1)		1.25	1.03–1.51*
25–34	1780 (39.0)	737 (37.2)		1.09	0.89–1.35
≥ 35	718 (15.7)	271 (13.7)		1.00	
Marital status			0.505		
Never married	253 (5.5)	92 (4.7)		0.84	0.62–1.13
Separated/divorced/widowed	509 (11.1)	225 (11.3)		1.01	0.82–1.25
Married	3806 (83.3)	1664 (84.0)		1.00	
Education			0.790		
No formal education	538 (11.8)	247 (12.5)		1.06	0.82–1.35
Primary	3067 (67.1)	1314 (66.3)		0.98	0.82–1.18
Secondary and above	963 (21.1)	420 (21.2)		1.00	
Occupation			0.313		
Employed	3135 (68.6)	1329 (67.1)		0.93	0.81–1.07
Unemployed	1433 (31.4)	652 (32.9)		1.00	
Parity			0.373		
Primigravida	1242 (27.2)	575 (29.0)		1.12	0.95–1.32
Secundigravida	936 (20.5)	417 (21.0)		1.08	0.89–1.29
Multigravida	2390 (52.3)	989 (49.9)		1.00	
Wealth index			0.886		
Rich	1484 (32.5)	656 (33.1)		0.98	0.84–1.15
Middle	888 (19.4)	374 (18.9)		0.95	0.79–1.16
Poor	2196 (48.0)	951 (48.0)		1.00	
Residence			0.251		
Urban	649 (14.2)	248 (12.5)		0.87	0.68–1.11
Rural	3919 (85.8)	1733 (87.5)		1.00	
Region			0.044*		
Northern	550 (12.0)	198 (10.0)		0.86	0.70–1.06
Central	1872 (41.0)	884 (44.6)		1.13	0.97–1.32
Southern	2146 (47.0)	899 (45.4)		1.00	
ANC visits			< 0.001***		
Adequate (≥ 4)	2017 (44.2)	1131 (57.1)		1.68	1.47–1.92***
Inadequate (< 4)	2551 (55.8)	850 (42.9)		1.00	
First ANC visit timing (*n *= 6413)			< 0.001***		
First trimester	985 (22.1)	518 (26.4)		2.72	1.97–3.76***
Second trimester	3065 (68.8)	1362 (69.6)		2.30	1.69–3.12***
Third trimester	405 (9.1)	78 (4.0)		1.00	

*ANC* antenatal care, *IPTp* intermittent preventive treatment for malaria during pregnancy, *CI* confidence interval, *OR* odds ratio

* *p* < 0.05; ** *p* < 0.01; *** *p* < 0.001

^a^*p* value of Pearson’s Chi squared test

### IPTp uptake stratified by number of ANC visits and timing of first ANC visit

Table 3 presents the odds of recommended IPTp uptake stratified by the number of ANC visits and timing of first ANC visit. After adjustment for age, parity, marital status, education, wealth, and occupation, women who had reported for the first ANC visit in their first and second trimesters were more likely to take the recommended IPTp dose (aOR = 1.97, 95% CI = 1.40–2.76 and aOR = 1.93, 95% CI = 1.42–2.64, respectively) than women who reported in their third trimester. Similarly, women with adequate ANC visits were more likely to take the recommended dose than those with inadequate ANC visits (aOR = 1.55, 95% CI = 1.34–1.80).

**Table 3:** Association between the number of ANC visits and IPTp uptake stratified by ANC timing

Timing of the first ANC visit	Number of ANC visit categories OR^a^ (95% CI)		Total
	Inadequate^b^	Adequate^b^	
First trimester	2.24 (1.44–3.49***)	3.41 (2.39–4.86***)	1.97 (1.40–2.76***)
Second trimester	2.19 (1.56–3.08***)	3.33 (2.36–4.70***)	1.93 (1.42–2.64***)
Third trimester	1.00	4.74 (1.61–13.96**)	1.00
Total	1.00	1.55 (1.34–1.80***)	

*ANC*  antenatal care, *IPTp* intermittent preventive treatment for malaria during pregnancy, *CI* confidence interval, *OR* odds ratio

* *p *< 0.05; ** *p* < 0.01; *** *p* < 0.001

^a^Adjusted for age, parity, marital status, education, wealth, and occupation

^b^The number of ANC visits was categorized into two levels: adequate ANC visits (four or more) and inadequate ANC visits (fewer than four)

Further examination of the association between the number of ANC visits and timing of first ANC visit (i.e., ANC use in the 6 groups) revealed that women with adequate ANC visits who had reported for their first ANC visit in the first or second trimester were approximately 3 times more likely to take the recommended IPTp dose (aOR = 3.41, 95% CI = 2.39–4.86 and aOR = 3.33, 95% CI = 2.36–4.70, respectively), than women who had inadequate ANC visits and had reported for their first ANC visit in the third trimester. Moreover, women with adequate ANC visits who reported for their first ANC visit in the third trimester were approximately 5 times more likely to take the recommended IPTp dose (aOR = 4.74, 95% CI = 1.61–13.96) than women in the reference group.

Notably, among women with inadequate ANC visits, those who had reported for their first ANC visit in the first and second trimesters were approximately 2 times more likely to take the recommended dose (OR = 2.24, 95% CI = 1.44–3.49 and OR = 2.19, 95% CI = 1.56–3.08, respectively) than reference group women.

The design of the MDHS survey incorporates both residents of the selected households and visitors. Thus, in the sample, only 58 women (0.9%) with a live birth 2 years before the survey were visitors. A sensitivity analysis to include only resident women for examination was further conducted and the results were consistent. Therefore, the findings from the whole sample were reported to comply with the MDHS survey design.

### Sub-group analysis for effects of socio-demographics on ANC attendance and recommended IPTp uptake

**Fig. 1:**
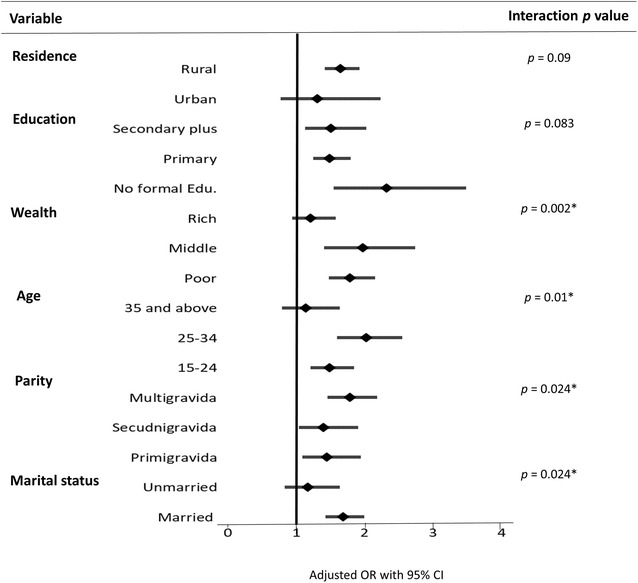
Sub-group analysis for effects of socio-demographics and number of ANC visits on recommended IPTp uptake. *p <0.05. The number of ANC visits was categorized into two levels: adequate ANC visits (4 or more) and inadequate ANC visits (fewer than 4) as reference. Results were adjusted for socio-demographic variables (i.e., age, parity, marital status, education, wealth, occupation, and timing of the first ANC visit, excluding the variable treated as the effect modifier)

**Fig. 2:**
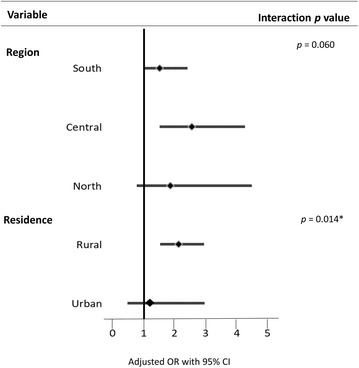
Sub-group analysis for effect of socio-demographics and ANC timing on recommended IPTp uptake. *p < 0.05. The timing of the first ANC visits was categorized into two levels: desirable timing (first and second trimester) and undesirable timing (third trimester) as reference. Results were adjusted for socio-demographics (i.e., age, parity, marital status, education, wealth, occupation, and number of ANC visits, excluding the variable treated as the effect modifier)

The interaction terms (i.e., number of ANC visits and socio-demographics (i.e., residence, education, wealth, age, parity, and marital status)) on IPTp uptake (*p* < 0.1) were considered in sub-group analysis. In the sub-group analysis, the effect of the number of ANC visits on IPTp uptake was significant in the poor and middle wealth groups (aOR = 1.94, 95% CI = 1.39–2.71 and aOR = 1.75, 95% CI = 1.45–2.11, respectively), 25–34 and 15–24 years old women (aOR = 2.02, 95% CI = 1.59–2.06 and aOR = 1.48, 95% CI = 1.19–1.83, respectively), all levels of parity with higher odds for the multigravida group (aOR = 1.74, 95% CI = 1.42–2.13), and married women (OR = 1.68, 95% CI = 1.42–1.98; Fig. 1). Furthermore, to examine ANC timing, reporting for the first ANC visit in the first and second trimesters (desirable ANC timing) was compared with reporting for the first ANC visit in the third trimester (undesirable ANC timing). The interaction between ANC timing and residence was significant (*p *= 0.014). The association between desirable ANC timing on IPTp uptake was significant only among rural residents (aOR = 2.13, 95% CI = 1.54–2.95; Fig. 2).

## Discussion

According to literature, this is the first study attempting to identify the association between timing of the first ANC visit and number of ANC visits, and also to evaluate the interactive effects of ANC attendance and socio-demographics on IPTp uptake. Having more ANC visits was expected to increase the likelihood of taking the recommended IPTp dose. However, the study revealed that although inadequate ANC visits (< 4) were noted, women who had their first ANC visit earlier in the first and second trimesters respectively had 2.24- and 2.19-fold increased odds of receiving the recommended IPTp dose. The effects of adequate ANC visits on taking the recommended IPTp dose were significant in married women, those aged ≤ 34 years, and those in the middle or poor wealth categories, whereas early ANC initiation was associated with an increased likelihood of taking the recommended IPTp dose among rural women only.

The rate of recommended IPTp uptake was 30.2%. A substantially lower rate (11%) was reported in Simuyu, Tanzania [44]. The change in the WHO IPTp policy in 2014 (minimum number of doses from 2 to 3) may have been partially responsible for this low uptake because most countries were still in the process of adopting the new guidelines. In the current study, only 48.1% of women had adequate ANC visits, similar to the 46% reported in an earlier Malawian study [45]. These results suggest that IPTp coverage and ANC attendance remain a challenge. The results increase the understanding of the factors affecting the uptake of such services, particularly emphasizing the associated effects of ANC attendance; further relevant research is warranted.

This study determined that women with adequate ANC visits were more likely to take the recommended IPTp dose, consistent with studies conducted in Cameroon and Ghana [31, 33]. This may be because IPTp policies in these countries recommend that IPTp should be administered at ANC visits, preferably as direct observation therapy (DOT). Consequently, women with higher numbers of ANC visits are more likely to complete the required IPTp doses. By contrast, the Kenyan study found no such association [29]. Other factors, such as availability of drugs at ANC clinics and effective DOT implementation, might also contribute to the observed differences [37, 46]. Additional studies are required to understand the health facility-based factors that could influence IPTp uptake, such as accessibility of drugs at clinics and availability of clean water for DOT.

The current study’s results support the findings observed in Tanzania and Ghana [23, 33], where women with ANC initiation in the first and second trimesters reported a higher IPTp uptake. However, other authors [34–37, 47] have reported no such association, potentially because of the different designs employed in these studies; for instance, Kibusi et al. [23] conducted a quantitative study, whereas Rass et al. [37] conducted a qualitative study. If a woman reports for ANC in the third trimester, she may not receive the full IPTp dose because of the delay in initiation of IPTp uptake.

Compared with other studies [23, 25, 26, 33, 38], this study further demonstrated the independent association between timing of the first ANC visit, in addition to the number of ANC visits, and IPTp uptake among pregnant women. In particular, the results suggest that reporting for the first ANC earlier during pregnancy, despite having an inadequate number of ANC visits, was associated with recommended IPTp uptake. ANC clinics are the main sources of disseminating crucial health information to women during pregnancy. Therefore, women may be advised on the importance of IPTp when they visit the ANC clinic early, and therefore, they may be able to source the drug from elsewhere if they fail to attend subsequent ANC visits [48].

Increased odds of recommended IPTp uptake in those with adequate visits despite having late initiation of ANC were also observed, but with a wider confidence interval, possibly because of the smaller sample number in this category. Two Ghanaian studies reported that 4 or more ANC visits were associated with higher IPTp uptake [26, 33]. The current findings further revealed the significance of this factor, independent of the timing of the first ANC visit, suggesting the need for healthcare workers to encourage late ANC initiators to make frequent ANC visits. However, women who report late for ANC with complications, such as malaria infection, may make frequent clinic visits and subsequently receive more SP as remedial treatment. In addition, the observed increased odds should be interpreted with caution because women who reported for ANC early in the third trimester may have completed the recommended IPTp dose, but those who reported late in the third trimester may not have.

Studies have reported age and marital status [23, 49], wealth [24, 30], education [23, 24, 30], and parity [23, 26, 30] to be significant predictors for IPTp uptake. However, this study did not observe a significant association between socio-demographics and IPTp uptake. Instead, moderating effects of socio-demographics on the association between ANC attendance and IPTp uptake were found in the sub-group analyses. Specifically, the effect of adequate ANC visits on taking the recommended IPTp dose was significant among married women, those in the poor and middle wealth groups, and women aged ≤ 34 years (*p *< 0.05). Notably, this relationship was significant at all levels of parity, with the highest odds observed for the multigravida group. In addition, the effect of desirable ANC timing on IPTp uptake was significant only among rural residents. Thus, tailor-made behavioural-change messages on IPTp uptake for different vulnerable groups of women are required to ensure effective scale-up of IPTp uptake.

The use of a nationally representative sample could strengthen the generalization of these results to Malawian women; however, because information on IPTp uptake and ANC attendance relied on recall, recall bias may have occurred. Recall ability is considered to be associated with the vividness, meaningfulness and significance of the event [50]. Recall may be better for less frequent and more significant events, such as the ones assessed in this study (IPTp uptake and ANC attendance), however, recall bias might still have compromised the findings. The MDHS data used for the analysis have not been verified with participants’ recalled information for either IPTp uptake or ANC attendance from the mothers on the ANC cards, which might have helped in reducing recall bias. Future studies should consider verifying recalled information by using the mothers’ ANC or health cards. Limiting the participants to those with a live birth in the 2 years preceding the survey also may have helped to reduce recall bias. Crucial factors, such as previous malaria infection, infection of family members, and health facility-based factors (i.e., drug stock-outs and ANC health worker attitude), which might affect pregnant mothers’ intention to return to ANC and thus their IPTp uptake, could not be evaluated in this study. Finally, because this was a cross-sectional study, causality could not be inferred.

## Conclusion

ANC attendance, namely the number of ANC visits and timing of the first visit, strongly influences IPTp uptake among Malawian women. The results demonstrated the independent association of these two ANC attendance assessments on IPTp uptake. In addition to frequent ANC visits, earlier ANC initiation should also be emphasized, which may simultaneously facilitate recommended IPTp uptake to appropriately protect pregnant women from the risk of malaria infection. The significant moderating effects of socio-demographics can facilitate the identification and targeting of vulnerable groups to promote the recommended IPTp uptake rate in Malawi. Further research is required to examine the effects of health facility-based factors on IPTp use and the effects of IPTp dose on birth outcomes. Qualitative studies interviewing both women and health personnel are also required for further elucidating barriers to recommended IPTp uptake.

## Abbreviations

IPTp: intermittent preventive treatment for malaria during pregnancy
MIP: malaria in pregnancy
ANC: antenatal care
MoH: Ministry of Health
WHO: World Health Organization
aOR: adjusted odds ratio
SP: sulfadoxine–pyrimethamine
DOT: direct observed therapy
DHS: demographic health surveys
MDHS: Malawi Demgraphic Health Survey
SEAs: standard enumeration areas
SPSS: Statistical Packages for Social Sciences
ICF: International Classification of Functioning Disability and Health
